# Biomechanical evaluation of natural tooth–implant splints and comparison with implant cantilevers in the anterior region under fatigue tests

**DOI:** 10.34172/joddd.025.39229

**Published:** 2025-06-30

**Authors:** Mahsa Karimoghli, Shima Ghasemi, Tahereh Ghaffari, Farhang Mahboub

**Affiliations:** Department of Prosthodontics, Faculty of Dentistry, Tabriz University of Medical Sciences, Tabriz, Iran

**Keywords:** Anterior region, Cantilever, Dental prosthesis, Fatigue test, Implant splint

## Abstract

**Background.:**

Several methods are used to replace lost teeth. This study aimed to reconstruct pre-maxillary conditions when a limited number of implants are available and investigate the biomechanics of the two methods available to dentists for use in these conditions (splinting natural teeth to implants and using a cantilever).

**Methods.:**

This in vitro study involved the preparation of eight bridge samples, which were divided into two groups. Four healthy recently extracted central teeth with similar sizes and a maximum difference of 20% in root and crown length were selected as the dental abutments. After preparing the samples, the temporal retention of Temp Bond cement was evaluated using a universal testing machine at a speed of 0.5 mm/min for both samples. A digital torquemeter was used to measure the torque required to open the abutments before and after a fatigue test. Data were analyzed using SPSS statistics software.

**Results.:**

A comparison of two types of prostheses before and after periodic loading showed that the amount of torque for loosening the abutment screw before applying force was the same in the two types of splint prostheses and cantilever prostheses. However, after applying a 200-N force, the amount of torque in the splinted prosthesis (19.75±1.70) was significantly higher than that of the cantilever prosthesis (12.1±5.73) (*P*<0.05).

**Conclusion.:**

Generally, dental implant prostheses exhibited better support in vitro compared to cantilever prostheses.

## Introduction

 Various methods are available for replacing lost teeth, with dental implants being a reliable option for intraoral reconstructions, even in challenging clinical conditions. However, the use of implants is not always ideal, particularly in the anterior maxilla, where surgical modifications may be necessary to enhance the success of the implant and improve the patient’s appearance and speech.^1,2^ Therefore, anatomical limitations have been reported, such as natural tooth intrusion, mechanical problems, caries, and implant placement or failure of osteointegration of one implant, prompting us to connect the implant to a natural tooth.^3^ The main problem with attaching an implant to a natural tooth is the different patterns of movement, which puts more stress on the implant.^4,5^ A growing number of studies have shown an increase in marginal bone loss or failure in implant osseointegration. Other problems are the loss of occlusal contact and failure of the abutment screw.^6^

 Attaching an ankylosed implant to an almost mobile tooth is not an ideal treatment.^7^ Despite the limitations of this treatment, some long-term clinical studies have not shown the destructive effects of implant attachment on teeth.^8-11^ The implants attached to natural teeth were acceptable for supporting fixed treatment prostheses, according to Belser et al.^12^ According to a review study by Shenoy et al,^2^ despite the conflicting results from implant-to-tooth attachment studies, in certain situations, the implantologist should consider implant-to-tooth attachment as an acceptable treatment option. In contrast, the loss of two adjacent teeth in the anterior maxilla or mandible has always presented a challenge for implant reconstruction. Due to the smaller diameter of the anterior teeth or the movement of surrounding teeth into the edentulous space, less space is needed to replace two implants.^13^ According to Tarnow et al,^14^ crystal bone resorption reduces the bone height between two implants when the space between them is < 3 mm. As a result, cosmetic problems and food entrapment will occur due to the disappearance of the papilla between the two implants. Using an implant cantilever seems to be an effective way to prevent such problems. No differences were found in a study by Hälg et al^15^ on bone resorption and implant survival in cantilever- and non-cantilever-prostheses.

 The results of various studies suggest that both tooth–implant splints and implant cantilevers may experience long-term problems. Static computer models are insufficient for predicting the long-term outcomes of treatment, and laboratory tests of fatigue are necessary for a realistic assessment. A challenge for dentists is achieving the ideal aesthetic results for anterior tooth implants, which may be hindered by anatomical and space constraints. Splinting natural teeth to implants and using a cantilever are strategies for addressing these limitations. This study aimed to reconstruct pre-maxillary conditions in cases where there is a limited number of implants and examine the biomechanics of these two methods that are available to dentists for use in these situations.

## Methods

**Figure 1:**
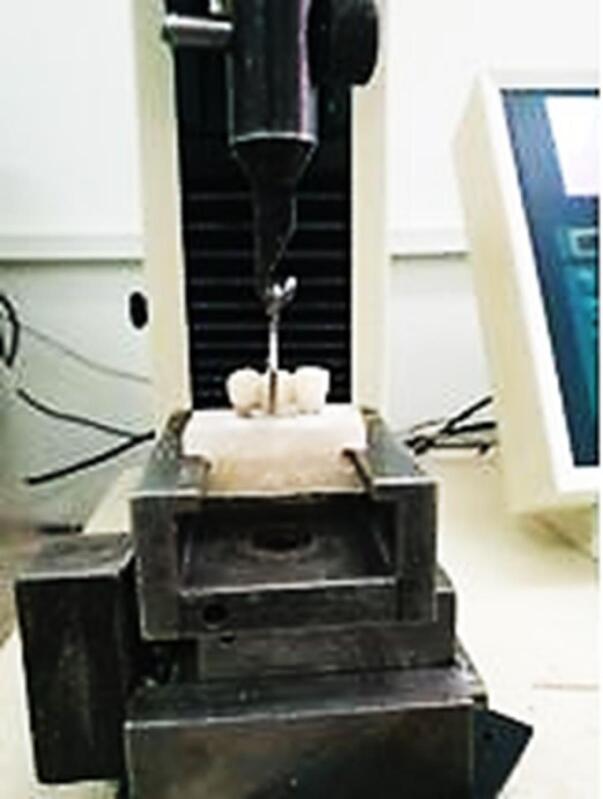
Pull-out test on a universal testing machine

 In this in vitro study, 8 bridge samples were prepared in two groups. The first group consisted of a three-unit porcelain fused to metal (PFM) with an implant–tooth base (sample A), and the second group comprised a two-unit cantilever bridge over the implant base (sample B). In this study, 8 three-piece root form implants (screw-retained, Internal hex) of 4 mm in diameter and 13 mm in length of Chaorum (MEDIMECCA, Seoul, Korea) with 8 straight abutments 4.5 mm in diameter and 7 mm in length, and 3 mm in the cuff of the same system were used. All the implants were buried in epoxy resin blocks at a distance of 3 mm apical from the block surface.^16^ Four newly extracted healthy central teeth of approximately the same size with a maximum of 20% difference in size along the root and crown length were selected for dental abutment in samples A. Synthetic periodontal ligament (PDL) around the central implants with a thickness of 0.5 mm and 1.5 mm below the cementoenamel junction (CEJ) around the roots was simulated using a polyester.^16^ The teeth were placed in epoxy resin blocks parallel to the implant at a distance of 5 mm from the implant. A surveyor was used to parallelize the samples during mounting. The abutments were closed on the implants with a 35-N torque using a digital torquemeter. The teeth were cut conventionally for metal–ceramic coatings. After dental mounts and implants, the samples were scanned with a UP3D 30 + scanner. A bridge with 10 × 7 mm PBX, 9 × 6 mm lateral, and 10 × 8 mm canine was designed by Exocad Galway 3 software. The amount of internal relief for cement space was considered 0.04 mm on all bases. To even out the shape and size of the metal frame and reduce the error of manual waxing, the frame pattern was printed by a printer (Digident Quik). Then, the alloy Ni-Cr (Wirobond C + , Bego Dental) was used to make the metal base. A porcelain–metal frame was used to prepare PFM coatings. To simulate the porcelain of the coating, one index coat was puttied, and the rest were pulverized by the index. After preparing the bridges, the coatings on the samples were cemented with Temp Bond NE cement. After preparing the samples, the temporal retention of Temp Bond cement was measured with a universal testing machine (Model HSK-S; Hounsfield test equipment, Surrey, UK) at a speed of 0.5 mm/min for both samples. In this way, the machine clamp was placed under the pontic in both samples, and the necessary force to remove the bridges was obtained in Newton. The initial retention value was recorded before the fatigue test. The pull-out test was repeated following the fatigue test (Figure 1).

**Figure 2:**
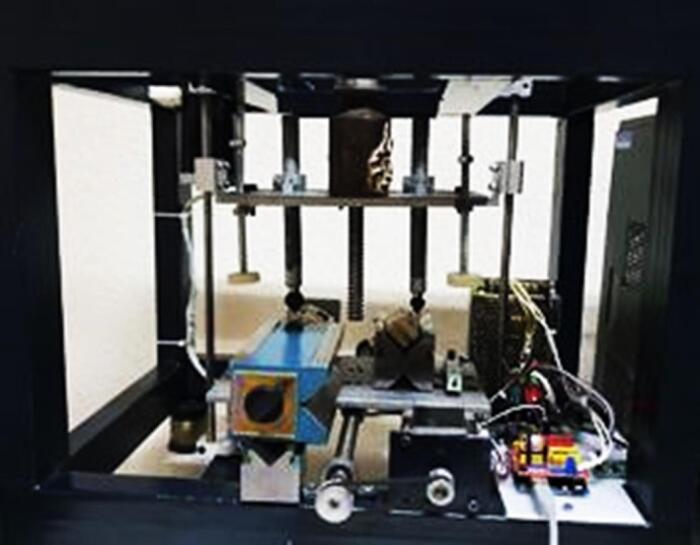
Fatigue test by Chewing Simulator CS-4

**Figure 3:**
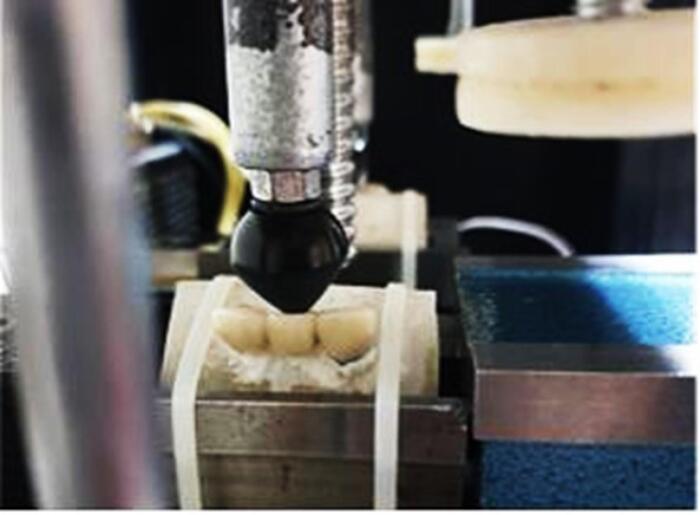
Forces on the bridge during the fatigue test

**Figure 4:**
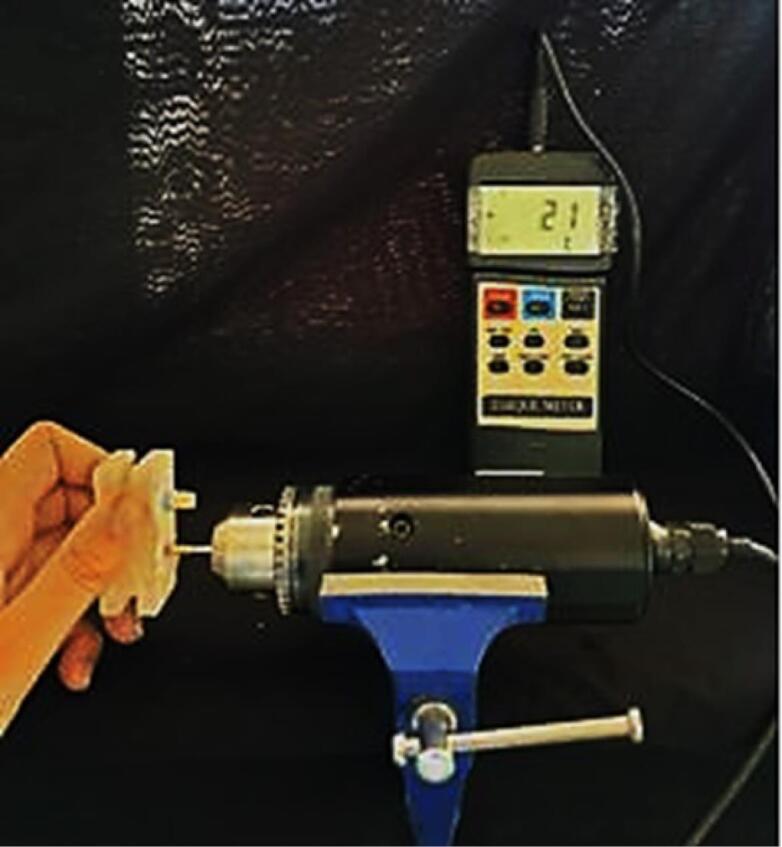
Measurement of abutment screw torque with a digital torque meter

 All the samples were placed in a chewing simulator (CS-4, SD-Mechanottronik) for fatigue testing. To simulate the anterior position of the mouth, forces were applied at an angle of 135º to the surface of the specimens.^17^ Cyclic forces of 200 N were applied to the specimens with a contact surface of 4 mm at the palatal surface at a distance of 2 mm from the incisal edge.^18,19^ The test frequency was set to 4 Hz because, according to articles, the frequency of human chewing is 1–4 Hz.^20,21^ The number of force cycles in each load was 10^6^ times. This is the number of swallowing and chewing actions in one year.^22,23^ After completing the fatigue test on all samples, the force required to remove the coatings was measured again with the universal testing machine at a speed of 0.5 mm/min. Following the fatigue test, the torque necessary for opening the abutments was measured using a digital torque meter (Figures 2, 3, and 4).

 SPSS 21 was utilized for data analysis. Given the small sample size, non-parametric tests were employed to meet the research objectives. The Mann-Whitney test was used to compare the two types of prostheses, and the Wilcoxon test was used to compare the results before and after force application.

## Results

 Periodic loading reduced the torque required for loosening the screw and decreased the tensile strength of both prostheses. A comparison of the two types of prostheses before and after periodic loading revealed that the amount of torque required for loosening the abutment screw before the application of force was similar for both splint prostheses and cantilever prostheses. However, after applying a 200-N force, the torque in the splinted prosthesis (19.75 ± 1.70) was significantly higher than that of the cantilever prosthesis (12.1 ± 5.73) (*P* < 0.05). Tensile strength before applying force was the same in two types of splinted prostheses and cantilever prostheses. After applying a 200-N force, the tensile strength in the splinted prosthesis (51.37 ± 6.47) was significantly higher than that of cantilever prostheses (28.47 ± 3.34) (*P* < 0.05) (Table 1).

**Table 1:** Comparison of the amount of torque required for loosening the abutment screw and tensile strength in two types of supported prostheses (implant–tooth and cantilever implant) before and after applying force

	**Prosthetic support** **type**	**No force applied**		**By applying force**		***P*** value**
		**Mean**	**SD**	**Mean**	**SD**	
The amount of torque to loosen the abutment screw	Implant–tooth	35.1	1.2	19.75	1.70	< 0.001
	Cantilever implant	35.8	1.5	12.5	1.73	< 0.001
	*P* value*	0.83		< 0.001		
Tensile strength	Implant–tooth	102.050	3.728	51.37	6.47	< 0.001
	Cantilever implant	94.400	8.228	28.47	3.34	< 0.001
	*P *value*	0.141		< 0.001		

* Mann-Whitney U test; **: Wilcoxon test.

## Discussion

 In this study, two common mechanical problems of dental implants were investigated, including loosening of the abutment screw and loss of cement traction of the coating during periodic loading in two fixed prosthesis support systems. In the present study, the cement strength of the coatings in both samples decreased significantly after applying periodic forces. Kaar et al^24^ showed the loss of cement retention in support implant prostheses under fatigue testing, so the rate of retention loss varied depending on the type of cement used and the force cycle. The researchers recommended Temp Bond cement because it is easy to remove the prosthesis in an emergency and has acceptable retention. The same cement was used in the present study. The results of the present study showed that the tensile strength in cantilever prostheses and implant–tooth splinted prostheses were similar before applying force. However, after periodic loading, the tensile strength in splinted prostheses was significantly higher than in cantilever prostheses. In some clinical situations, the use of a cantilever is the most conservative treatment option; however, due to the creation of a lever arm, the cantilevers cause a disproportionate increase in force on the implants, abutment screws, cement, and the bone–implant contact surface.^13^ Numerous studies have shown that the concentration of stress and pressure in cantilever-supporting implants is higher than in non-cantilever implants.^25^

 It has also been reported that this pressure is mainly concentrated in the alveolar bone crest and adjacent to the distal surface of the implant to which the cantilever prosthesis is attached.^26^ However, another group of studies emphasizes the practical and clinical success of implant-based cantilever prostheses and believes that there is no significant difference in the performance of these prostheses compared to non-cantilever prostheses.^15^ According to Greenstein et al,^27^ cantilever prostheses act as a type 1 lever, and the forces operating on the cantilever produce 2–3 times more stress than supported prostheses on both sides. A review study by Pjetursson et al^28^ showed that the survival rate of fixed cantilever prostheses after 5 years was 91.4%, and in fixed prostheses with implant-dental support, it was 95.5%. These results, consistent with the present study, showed a higher survival rate of prostheses with implant–tooth support. However, the studies of these researchers showed that during ten years, the survival rate of cantilever prostheses (80.3%) was slightly higher than that of prostheses with implant–tooth support (77.8%). Zurdo et al^25^ also showed that cantilever prostheses suffer twice as many complications as non-cantilever prostheses after 5 years.

 The study of Mokhtari et al,^26^ contrary to the results of the present study, indicated no significant relationship between the presence or absence of a cantilever and the rate of bone resorption, but the rate of bone resorption was related to the time factor. Rammelsberg et al^29^ also showed that the failure of implant-based and implant–tooth prostheses have no significant relationship with the type of prosthesis support. Becker^30^ showed the success of implant-based cantilever prostheses over ten years. These researchers stated that the problems with denture-based cantilever prostheses should not be attributed to these prostheses. However, the long-term results of implant-based cantilever prostheses are not fully known. A review study by Shenoy et al.^2^ showed various complications for implant-supported prostheses due to intrusion and overloading of the implant, which causes loss of marginal bone associated with overload around the implant.

 Although no comparison has been made between the two types of implant-based fixed prosthesis support systems in these studies, each has examined the strengths and weaknesses of these systems separately. Reasons for differences in study results include duration of prosthesis survival, prosthetic material, tooth type, jaw type, age, and gender. Also, the laboratory and clinical nature of the studies and the physical properties and viscoelastic behaviors of the PDL in clinical studies, especially in tooth–implant splints, are the reasons for the disagreements between various articles. Davis et al^31^ named many anatomical and biological risk factors for implant attachment to the tooth. One of the problems is normal tooth movement along the PDL, as a force of 0.1 Newtons leads to a movement of 50–200 micrometers in the tooth, while a rigid implant moves only 10 micrometers. Therefore, the pattern of stress and strain distribution in the bone around the implant and the tooth following chewing is different, and this can lead to the failure of prostheses with implant–tooth support in the long run.

 Another common problem is the loosening of the abutment screws. Fatigue due to periodic loading causes preload to be lost and, eventually, the screw to loosen. In the present study, the torque required to unscrew the abutment was reduced in both types of samples after loading, but the rate of reduction was greater in cantilever samples. The results also showed that before applying force, the torque required to unscrew the abutment was similar in both types of prostheses; however, after periodic loading, the amount of torque in cantilever prostheses was significantly less than that in splinted prostheses.

 Any inconsistency in occlusion, matching of the form, or forces can cause the screw to loosen or break during operation. Reports indicate that 6–20% of maxillary prostheses undergo loosening of the screw at least once in the first year of operation.^10^ Cantilevers are force intensifiers and represent a significant risk factor for the weakening of screws, crystal bone resorption, fractures, and any other factor that is negatively affected by force. Zurdo et al^25^ stated that the main complaint of cantilever prostheses is the loosening of the abutment screw and the breaking of porcelain. Kourtis et al^32^ considered the most common problem of implant-supported prostheses to be the loosening of the abutment screw and introduced aggravating factors such as oral parafunctional habits, type of restoration, and cantilever. Studies have shown that despite the higher probability of technical problems such as loosening of the abutment and screw or therapeutic complexities, the cantilever treatment plan can be considered a valid and sustainable method.^33^

## Conclusion

 In general, tooth–implant prostheses are better supported in vitro than cantilever prostheses, and it appears that the destructive effect of natural tooth movement inside the PDL space is less than the lever effect of cantilever prostheses, and cover retention and abutment screw torque are less affected.

## Competing Interests

 The authors declare no conflicts of interest.

## Ethical Approval

 The present research was approved by the Ethics ID IR.TBZMED.VCR.REC.1399.286 in the Research Ethics Committee of Tabriz University of Medical Sciences.
